# Effect of Enhanced Gravity on the Microstructure and Mechanical Properties of Al_0.9_CoCrFeNi High-Entropy Alloy

**DOI:** 10.3390/e22111318

**Published:** 2020-11-19

**Authors:** Anjun Shi, Ruixuan Li, Yong Zhang, Zhe Wang, Zhancheng Guo

**Affiliations:** 1 State Key Laboratory of Advanced Metallurgy, University of Science and Technology Beijing, Beijing 100083, China; b20160501@xs.ustb.edu.cn (A.S.); zhewang@ustb.edu.cn (Z.W.); 2 State Key Laboratory for Advanced Metals and Materials, University of Science and Technology Beijing, Beijing 100083, China; b20180468@xs.ustb.edu.cn (R.L.); drzhangy@ustb.edu.cn (Y.Z.)

**Keywords:** enhanced gravity, microstructure, high-entropy alloys, mechanical properties

## Abstract

The influence of enhanced gravity on the microstructure and mechanical properties of the Al_0.9_CoCrFeNi high-entropy alloy, which was solidified under normal gravity (acceleration 1 g) and enhanced gravity (acceleration 140 g, acceleration 210 g, and acceleration 360 g) conditions is reported in this paper. Its solidification under enhanced gravity fields resulted in refinement of the columnar nondendritic grain structure and an increase in the area fraction of the body-centered cubic (BCC) structure phases. The mass transfer strengthened by enhanced gravity promoted element diffusion and enrichment, which caused changes in the composition and microstructure that, in turn, affected the mechanical properties of the alloy. The compressive strength and plasticity of the sample solidified at acceleration 360 g were equal to 2845 MPa and 36.4%, respectively, which are the highest values reported to date for Al_0.9_CoCrFeNi alloy.

## 1. Introduction

High-entropy alloys (HEAs), a new family of metal materials, have received great attention in recent decades [1,2,3,4,5]. Their design strategies differ from those of traditional alloys due to their enhanced mixing entropy [6,7]. As a result, random yet stable solid-solution phases often form instead of ordered intermetallic phases. HEAs exhibit high strength, good ductility, excellent wear, and corrosion resistance [8,9,10], which make them strong potential candidates for structural materials for a variety of applications [11,12,13].

Al_x_CoCrFeNi (with x = 0–1) is one of the most extensively studied HEAs and possesses high impact resistance and strength [14,15,16]. When small amounts of Al are present in the alloy (x < 0.5), the alloy crystallizes in a face-centered cubic (FCC) symmetry. The resulting material possesses relatively low strength, high ductility, and significant work hardening ability. At x = 0.5–0.9, the alloy hardness is higher because of the presence of a body-centered cubic (BCC) structure phase. At higher Al contents, only BCC crystals form, and the resulting material demonstrates high brittleness and strength.

The Al_0.9_CoCrFeNi alloy, containing an FCC and BCC dual structure, has been extensively studied [17,18,19]. Dendrites are present in the microstructure, which provide the material with outstanding strength and plasticity [20]. Further improvements in the mechanical properties of the Al_x_CoCrFeNi alloy using various treatment methods have also been extensively studied, including heat treatment methods [21,22], alloying element addition [14], and thermo-mechanical treatment [23]. However, few studies have provided a systematic and comprehensive analysis of Al_x_CoCrFeNi HEA solidification, and even fewer have improved the mechanical properties via controlling its solidification process. Although the refinement mechanism remains controversial, it is known that solidification of an alloy in enhanced gravity fields often leads to grain refinement, thus improving its mechanical properties [24,25]. The combination of enhanced gravity and combustion synthesis methods has been successfully used in the preparation of various series of HEAs [26]; however, the feasibility of applying enhanced gravity to improve the mechanical properties of HEAs has not been reported and remains questionable.

Therefore, we constructed a phase diagram for the Al_x_CoCrFeNi HEA, systematically analyzed its solidification process, and proposed a new method for its fabrication using enhanced gravity. Unlike conventional procedures (e.g., vacuum solidification), this technology can directly change the solidification environment, significantly shorten the processing time, and reduce the energy consumption. We also studied how the acceleration value affected the mechanical properties of the Al_0.9_CoCrFeNi alloy.

## 2. Materials and Methods

The Al_0.9_CoCrFeNi alloy was prepared in a metal mold using a vacuum induction furnace. It was machined into a rod, 26 mm in diameter and 30 mm long, for consequent enhanced gravity experiments. The initial alloy composition is shown in Table 1.

**Figure 1:**
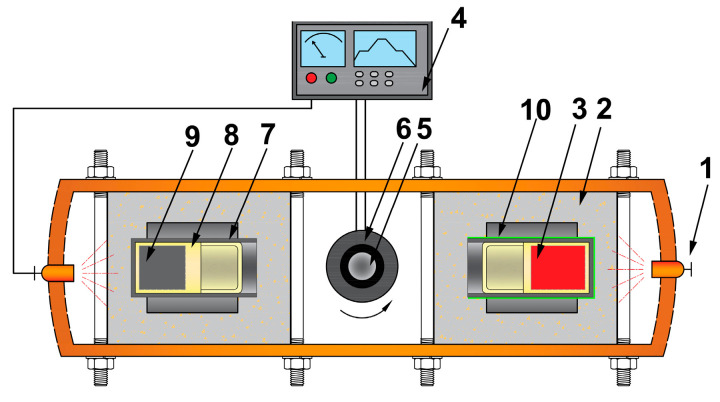
Schematic of the experimental apparatus: (1) microwave generator; (2) thermal insulator; (3) Al_0.9_CoCrFeNi alloy sample; (4) temperature-controlling system; (5) centrifugal axis; (6) conductive slip ring; (7) graphite crucible; (8) alumina crucible; (9) counterbalance sample; (10) microwave absorbing material.

The enhanced gravity field was obtained using a centrifuge apparatus (see Figure 1). The sample was placed into an alumina crucible, which was inserted into a graphite crucible—the external walls of which were coated with the microwave-absorbing material capable of withstanding 1600 °C. This microwave-absorbing material can absorb the microwave and provide heat to the sample. Then, this graphite crucible was positioned horizontally in one thermally insulated cavity. On the opposite side, another graphite crucible without the microwave-absorbing coating was filled with the same amount of sample and then loaded into another thermal insulator to keep balance of the centrifugal apparatus. Once the sample was heated to the target temperature, the centrifugal system was started.

To evaluate the enhanced gravity field values, we used the concept of acceleration value, the acceleration value is *x* g, and the expression of acceleration value coefficient *x* is defined as a ratio of the centrifugal and the normal gravity accelerations (see Equation (1)).

(1) $$x=\frac{\sqrt{g^{{}^{2}}+{\left(\omega^{2}r\right)}^{2}}}{g}=\frac{\sqrt{g^{2}+{\left(\frac{N^{2}\pi^{2}r}{900}\right)}^{2}}}{g}$$

where *N* is the centrifuge rotation speed (rpm), *ω* is the angular velocity (rad/s), *r* is the distance from the centrifugal axis to the sample (0.27 m in this study), and g is the normal gravitational acceleration (9.8 m/s^2^).

During the enhanced gravity solidification experiments, the sample rod was heated at 1600 °C (ramped at 26 °C/min rate) for 40 min until it melted, after which the centrifuge was turned on and operated at angular velocities equal to 700, 850, and 1120 rpm. These acceleration values corresponded to acceleration 140 g, acceleration 210 g, and acceleration 360 g, respectively. The sample was then cooled to 1100 °C at 15 °C/min. At this temperature, solidification of the Al_0.9_CoCrFeNi alloy occurred. Reference samples were obtained using the same method but under normal gravity (acceleration 1 g) conditions.

**Figure 2:**
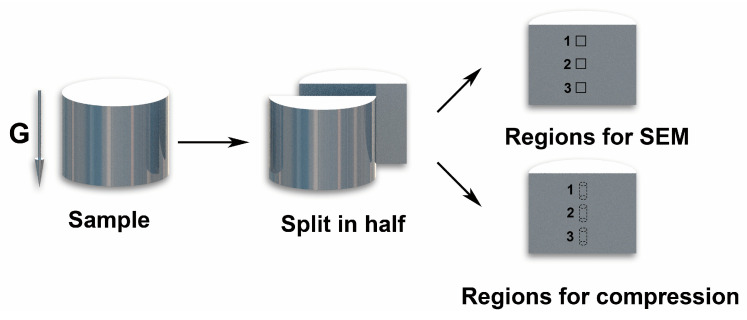
Sample preparation for scanning electron microscopy (SEM) and compression tests, as well as the corresponding points of the measurements.

The phase compositions were determined using X-ray diffraction (XRD). The microstructures of the samples were characterized by scanning electron microscopy (SEM) performed using the FEI Quanta-400F instrument. The chemical composition of the original Al_0.9_CoCrFeNi HEA was analyzed by Inductively Coupled Plasma-Optical Emission Spectrometry (ICP-OES, Optima 7000DV, Perkin Elmer, MA, USA). The distribution of micro chemical composition of Al_0.9_CoCrFeNi HEA was obtained by an electron probe micro-analyzer (EPMA-1720, SHIMADZU, KYOTO, JAPAN), and the content of each element is the average of nine regions to ensure the accuracy of the results. Electron backscattered diffraction (EBSD) was performed at 20 kV using an 18 mm working distance and a 70° tilt angle. The area fraction of different phase in the Al_0.9_CoCrFeNi alloy was calculated by Image-pro Plus software 6.0 (Media Cybernetics, Silver Spring, MD, USA) Room temperature compressive tests were performed using cylindrically shaped samples (3 mm in diameter and 6 mm in length) using an MTS809 machine under a 1 × 10^−3^ s^−1^ strain rate. The surface roughness of the compression sample is lower than Ra0.8., and the lubricant is evenly coated on the surface. The phase equilibrium diagrams were constructed using the PanHEA thermodynamic database. The dimensions and shapes of the samples used for the SEM and compression tests are shown in Figure 2. The cylindrical sample was cut in half, and each of the halves was then used for further tests. The SEM and compression tests were performed along the cylinder center axis and the enhanced gravity field axis.

## 3. Results

### 3.1. Phase Composition and Microstructure

**Figure 3:**
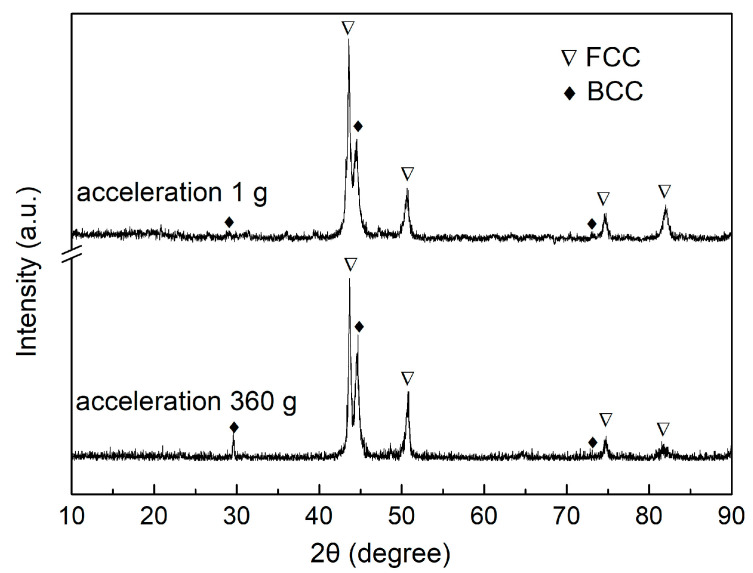
X-ray diffraction (XRD) patterns of the Al_0.9_CoCrFeNi alloy prepared at acceleration 1 g and acceleration 360 g. FCC, face-centered cubic structure; BCC, body-centered cubic structure.

The XRD patterns of the samples prepared at acceleration 1 g and acceleration 360 g are compared in Figure 3. The strongest features were peaks corresponding to the BCC and FCC structure, thus, both phases formed under both normal and enhanced gravity conditions. Furthermore, the three strong peaks of the BCC structure become stronger when acceleration 360 g, because the content of BCC structure phases increase during the enhanced gravity-affected solidification process. The BCC structure phases include disordered B1 phase and ordered B2 phase. It is revealed by other reports that the lattice constants of the B1 and B2 phases in the AlCoCrFeNi alloy are close, so the corresponding diffraction peaks are difficult to distinguish [20].

**Figure 4:**
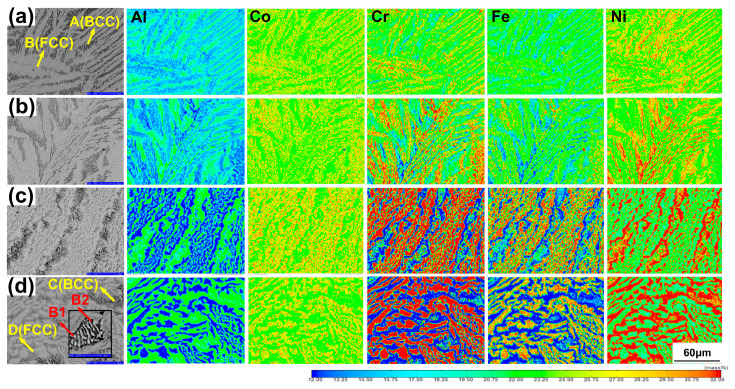
Back scattered electron (BSE) images and EPMA elemental mapping of the Al_0.9_CoCrFeNi alloy obtained at (**a**) acceleration 1 g; (**b**) acceleration 140 g; (**c**) acceleration 210 g; (**d**) acceleration 360 g. Here, the grey regions are the FCC phase and the black regions are the BCC structure phases. In (**d**), coexisting B1 and B2 phases are enlarged in the inset.

Figure 4 shows backscattered electron (BSE) images and the corresponding EPMA elemental mapping performed on samples obtained under different acceleration values. Table 2 lists the average contents of the BCC and FCC structure phases and the accurate contents of B1 and B2 phases in the samples obtained at acceleration 1 g and acceleration 360 g, which is marked in Figure 4d. Columnar nondendritic structures were observed in all samples (see Figure 4a–d). The grey regions, marked as “B” and “D” in Figure 4a,d are the side plates with an FCC structure. FCC structure phases consist primarily of Fe and Cr, while the amount of Al and Ni is smaller there. The black regions, marked as “A” and “C” in Figure 4a,d, contain a mixture of B1 and B2 phases. B2 phase is rich in Al and Ni (containing more lighter elements with black), and B1 phase consists mainly of Cr (and next Fe) and with small concentrations of Al and Ni (containing more heavier elements with white), as has been enlarged in the inset in Figure 4d. It can be seen from Figure 4 that, when acceleration was 210 g and acceleration was 360 g, the total amount of B1 and B2 phases increase, as this is compared to an acceleration of 1 g. The side plates of the FCC structure phase obtained under enhanced gravity fields were finer in samples obtained at acceleration 1 g. The thinnest side plate structure was obtained at acceleration 360 g.

As the acceleration value increased, the aggregation of the main alloy elements became more apparent (see Table 2). A higher Al and Ni content in the B2 phase increased to 19.48% and 35.91% at acceleration 360 g. (from 11.35% and 24.00% at acceleration 1 g). At the same time, the Cr content in the B1 phase increased slightly to 22.81%. (from 22.54% at acceleration 1 g). The Co, Cr, and Fe elements are demonstrated to aggregate in the FCC phase, respectively, at acceleration 360 g. It should be noted that all the samples represent homogeneity inside the grains (Figure 4a–d).

**Figure 5:**
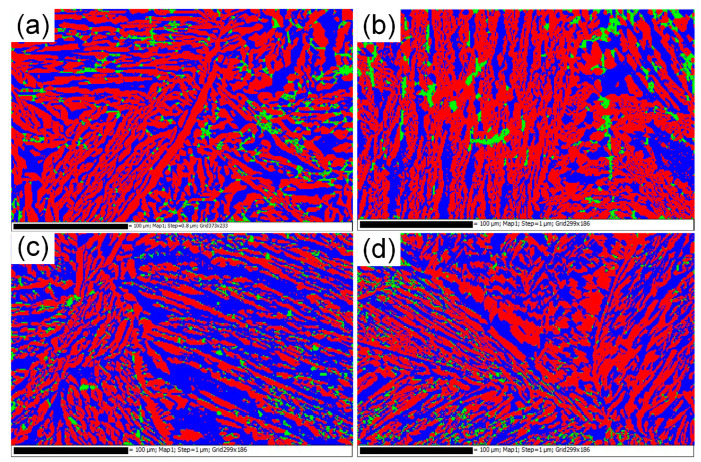
Electron backscattered diffraction (EBSD) data for the Al_0.9_CoCrFeNi alloy samples obtained under (**a**) acceleration 1 g; (**b**) acceleration 140 g; (**c**) acceleration 210 g; (**d**) acceleration 360 g. The red-, blue-, and green-colored areas correspond to the FCC, BCC, and unrecognized structure phases, respectively.

Figure 5 shows the microstructure, grain morphology, and phase distribution (all obtained by EBSD) of the samples subjected to various enhanced gravity fields. The blue and red areas represent the BCC and FCC structure phases, respectively. Note that the EBSD cannot differentiate B1 and B2 phases, but it can be used to count the total area fraction of B1 and B2 phases. There is a small part of area that cannot be recognized by EBSD, which is presented in green. It is worth noting that the small proportion and sporadic distribution of sigma phase with the same composition as B1 phase cannot be excluded in the green area (Figure 5). As the acceleration value increased, the sample microstructure became more refined and the width of the lamellar features became smaller. A significant increase in the area fraction of BCC structure phases were also observed (from 32.3 to 58.9%) as the acceleration value was increased from acceleration 1 g to acceleration 360 g.

### 3.2. Mechanical Properties

**Figure 6:**
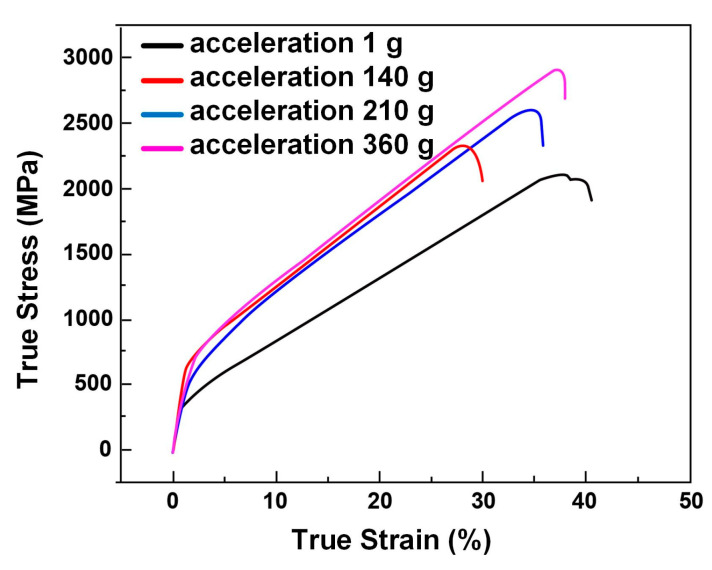
Compressive stress–strain curves of the Al_0.9_CoCrFeNi alloy samples prepared at different acceleration values: acceleration 1 g, acceleration 140 g, acceleration 210 g, and acceleration 360 g.

The room temperature true compressive stress–strain curves obtained using a 1 × 10^−3^ s^−1^ strain rate are shown in Figure 6. The average (out of three measurements performed, as shown in Figure 2) specific values of the compressive strength and plasticity are listed in Table 3. Under enhanced gravity, the specimen shows mixed fracture of 0° and 45° directions after compression fracture, while the specimen shows 45° fracture under normal gravity. The alloy’s plasticity decreased from 38.2% to 27.9% as acceleration value increased from acceleration 1 g to acceleration 140 g. However, at higher acceleration values, the plasticity with acceleration 360 g was close to but still lower than acceleration 1 g. At the same time, the compressive strength increased linearly as the acceleration value increased and reached its maximum at acceleration 360 g. The behavior of the plasticity and compressive strength is directly related to the microstructure refinement and to the increased BCC structure phases fraction at high acceleration values (see Figure 6).

## 4. Discussion

### 4.1. Phase Transition in the Al_0.9_CoCrFeNi HEA Samples

**Figure 7:**
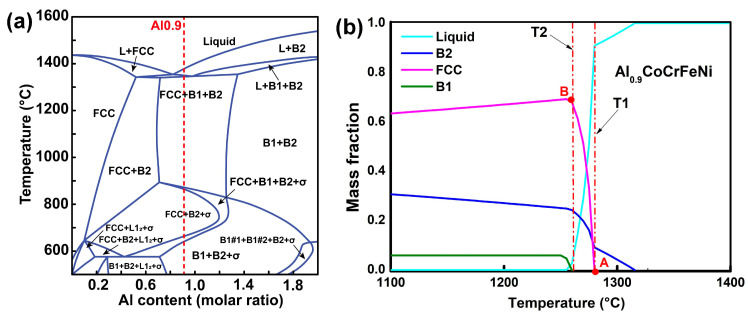
Equilibrium phase diagrams calculated using the PanHEA database: (**a**) phase compositions at different temperatures as a function of Al content in the alloy; (**b**) phase compositions at different temperatures for Al_0.9_CoCrFeNi. The red dashed line in (**a**) shows the (**b**) cross-section in relation to the phases of the Al_x_CoCrFeNi alloy with x = 0–2.0.

The microstructure of the Al_0.9_CoCrFeNi HEA observed in this work is consistent with the equilibrium phase diagram obtained using the PanHEA database (see Figure 7a). Figure 7b reflects the relationship between the mass fraction of the different phases and the temperature for the Al_0.9_CoCrFeNi alloy. As the temperature decreases, the primary B2 phase (rich in Al-Ni) starts to nucleate at the liquidus temperature. When the temperature drops to T1 (1280 °C), the FCC and B2 phases precipitates from liquid alloy simultaneously at point A. When the temperature reaches T2 (1260 °C), the eutectic reaction occurs at point B and the liquid phase disappeared completely. Below the temperature of T2 (1260 °C), only the solid phases (FCC, B2, B1) exist in the system, and the solid-state phase transition from B2 to B1 occurs at this stage. The lattice constants of B2 and B1 phases have very little difference and coexist in the crystal, as shown in Figure 3. In the solid-phase phase transition, the B2 phase forms a connecting matrix-like network, while the B1 phase (rich in Cr) forms isolated domains inside this matrix [27]. In this study, we applied enhanced gravity with the HEA sample cooled from 1600 °C to 1100 °C. In fact, enhanced gravity field only has an effect on the solid–liquid coexistence zone; that is to say, in the liquid-B2 coexistence zone from 1600 °C to 1280 °C and in the liquid-B2-FCC coexistence zone from 1280 °C to 1260 °C. Then, continuing to solidify from below 1260 °C, only solid phases (FCC, B2, B1) exist in the system, and the enhanced gravity has nearly no effect at this stage.

### 4.2. Microstructure and Mechanical Properties

The main theories state that the phase and grain refinement occurs as a result of (1) the nucleation energy change [28], (2) the faster cooling rates [29], (3) the dendrite fragments, [24] (4) as well as their coexistence with free-chill crystals [30]. It was recently demonstrated that the enhanced gravity field strengthens the crystal nucleation to the extent of “crystal rain,” which significantly accelerates crystal nucleus migration [25]. Mass transport could also be significantly enhanced under the enhanced gravity field due to gravity-induced convection [31]. Thus, high centrifugal pressure might also make the element diffusion rates faster.

In addition to the increase in heterogeneous nucleation cores caused by enhanced convection, it is proposed that the critical nucleation energy will be reduced by enhanced gravity. When liquid metal begins to nucleate, the nucleation energy Δ*G* is mainly composed of two parts under constant gravity, one is the difference of volume free energy, and the other is the increase in interface energy Δ*G_i_*.

(2) $$\Delta G=\Delta G_{V}+\Delta G_{i}=\Delta G_{m}V+\sigma_{1s}A$$

Wang et al. [32] studied the effect of enhanced gravity on the solidification structure of Al-Cu alloy. The pressure of the system changes due to enhanced gravity, which affects the change of nucleation energy. The formula of nucleation energy can be shown in Equation (3).

(3) $$\Delta G^{*}=\frac{16}{3}\cdot \frac{\pi \sigma_{1s}^{3}}{{\left(\Delta F_{T}-\frac{K\varepsilon \rho \omega^{2}r^{2}}{2}\right)}^{2}}$$

where Δ*F_T_* is the change in the chemical potential of the solidification system at temperature *T*, *r* is centrifugal radius, *σ*_1*s*_ is interfacial tension, ε is body contraction rate, *K* is conversion factor, and *ω* is rotational angular velocity.

It can be seen from Equation (3) that *r* remains unchanged in the experiment, and the system chemical potential Δ*F* is negative during solidification. Increasing the rotation speed *ω* leads to an increase in the acceleration value, a smaller critical nucleation work, easy formation of crystal nuclei, and the effect of grain refinement. Even though, the scientists still debate the grain refinement mechanisms occurring under the enhanced gravity application.

Our samples were subjected to a strong centrifugal force and solidified under enhanced gravity fields. The centrifugal pressure *P_i_* inside the melt under enhanced gravity field can be expressed as [31]:

(4) $$P_{i}=\frac{\rho \omega^{2}\left(r_{i}^{2}-r_{0}^{2}\right)}{2}$$

where *ρ* is the density of the liquid alloy (equal to 6.1 g·cm^−3^ for Al_0.9_CoCrFeNi), *r*_0_ and *r_i_* (equal to 0.24 and 0.27 m, respectively) are distances from the bottom surface of the sample and from point *i* (located at the sample top surface) to the rotation axis, respectively. Thus, in our case, the centrifugal pressure of the HEA melt can be expressed as:

(5) $$P_{i}=1.73\times 10^{3}\sqrt{x^{2}-1}$$

At acceleration values equal to acceleration 140 g, acceleration 210 g and acceleration 360 g, *P_i_* values were equal to 0.24, 0.36, and 0.62 MPa, respectively. We also used the Clapeyron equation, shown below:

(6) $$dT=\frac{T_{m}\left(V_{L}-V_{S}\right)}{\Delta H}dP$$

where d*T* and d*P* refer to the change in the melting point and pressure, respectively; *V_L_* and *V_S_*. are the specific volumes of the liquid and solid phases, respectively; Δ*H* and *T_m_* are the latent heat of fusion and the corresponding melting point, respectively.

According to our phase diagram, first, the B2 phase separates from the liquid phase upon cooling. At the temperature T2 (1260 °C), the eutectic reaction occurs. The densities of the Al_0.9_CoCrFeNi melt, the B2, and the FCC phases are 6.32, 5.91, and 7.23 g·cm^−3^, respectively, according to the PanHEA database. As the specific volume is the inverse of the density, we can infer that (*V_L_* − *V_B_*_2_) < 0 and (*V_L_* − *V_FCC_*) > 0. Taking into account Equation (6), we concluded that the melting point of the FCC phase increases under high pressure, while the opposite occurs for the B2 phase. Therefore, high centrifugal pressure could contribute to the formation of the FCC phase and the disappearance of the primary B2 phase in the Al_0.9_CoCrFeNi alloy, which shifts the eutectic point to higher Al contents (to the right in the phase diagram). As the Al_0.9_CoCrFeNi alloy is a hypereutectic alloy, this shift can reduce the FCC phase fraction (according to the leverage theorem), which is consistent with the SEM results, which showed increased B2 contents at high acceleration values.

B2 phase is the primary precipitated phase, which is rich in Al. Under the environment of enhanced gravity, due to the density difference between B2 phase and liquid phase (5.91, and 6.32 g·cm^−3^), solid B2 phase moves to the surface of liquid phase of Al_0.9_CoCrFeNi alloy. Note that the mixing enthalpy of the Al and Ni is negative and there is a strong mutual attraction, so the Ni in the molten liquid is attracted to the B2 phase, and this process is enhanced due to the presence of enhanced gravity. Under enhanced gravity, Al and Ni elements diffuse sufficiently to the region far away from the crystallization front, so that the concentration of the crystalline liquid gradually increases, which causes the final B2 phase to be more enriched in Al and Ni. On the basis of the elements, diffusion is intensified under enhanced gravity in the liquid-B2 coexistence zone from 1600 to 1280 °C and in the liquid-B2-FCC coexistence zone from 1280 to 1260 °C, the diffusion of Cr is slightly accelerated enriched in the solid phase transition of B2 to B1 below 1260 °C.

In fact, the total area fraction of the BCC structure phases calculated in Figure 5 is the area fraction of the B1 and B2 phases. The knowledge of stoichiometry of the phases occurring in a sample and the knowledge of its nominal composition allowed to determine the actual phase composition of this sample, by minimizing the summation value [15].

(7) $$S=\sum_{j}(\sum_{i}p_{i}x_{ij}-n_{j}{)}^{2}$$

where *p_i_* denotes the amount of *i*-th phase, *x_ij_* stands for the concentration of *j*-th element in the *i*-th phase and *n_j_* denotes the nominal concentration of element *j*. Such calculated data sources were performed from Table 1 and Table 2. The detailed amounts of B1, B2 and FCC phases can be obtained, being compared with the area fraction of BCC phases in Figure 5. According to Equation (7), when acceleration 1 g, the mass fraction of B1 and B2 phases are 5.1% and 14.7% (total 19.8% BCC structure phases), respectively. Similarly, when acceleration 360 g, the mass fraction of B1 and B2 phases are 15.1% and 41.2% (total 56.3% BCC phase), respectively. Convert the area fraction of the BCC structure phases calculated in Figure 5 into mass fraction as a total of 21.2% when acceleration was 1 g and 57.6% when acceleration was 360 g. Using minimizing the summation value, it is good agreement with the corresponding amount of total BCC structure phases determined using EBSD data. It can be seen from the above discussion that once the acceleration value increases, the B2 phase generation increases by the eutectic reaction, which promotes the increase in B1 phase content in the system due to the solid phase transition. However, the main contribution of the increase amount of BCC phase comes from the B2 phase at room temperature.

Table 4 compares the ultimate compressive strength and plasticity of the Al_0.9_CoCrFeNi HEA under the enhanced gravity field with other traditional Al_x_CoCrFeNi HEAs (such as Al_0.5_CoCrFeNi, Al_0.75_CoCrFeNi, Al_0.9_CoCrFeNi, and AlCoCrFeNi). The mechanical properties of Al_x_CoCrFeNi HEAs vary significantly depending on their processing conditions. The compressive strength and plasticity of the traditional dual-phase Al_x_CoCrFeNi alloy prepared by melting, casting, and enhanced gravity combustion were <2000 MPa and >20%, respectively. The AlCoCrFeNi HEAs fabricated by the additive manufacturing also show a great mechanical property, while the compressive strength of them still smaller than 2000 MPa. On the contrary, the compressive strength of our alloy processed at acceleration 360 g reached 2845 MPa and maintaining 36.4% plasticity. In order to further improve the plasticity, a higher enhanced gravity coefficient is expected in our future work. Thus, the ultimate compressive strength and plasticity values of our Al_0.9_CoCrFeNi alloy exceed those of other dual-phase Al_x_CoCrFeNi alloys. Such excellent properties are attributed to the microstructure refinement and high content of the hard BCC structure phases (including B1 and B2 phase). Additionally, compared with the equal-atomic Al_x_CoCrFeNi alloy containing only the BCC phases, our alloy also had a similar compressive strength and much better plasticity because of the presence of the ductile FCC phase.

## 5. Conclusions

(1) Al_0.9_CoCrFeNi alloys processed under normal and enhanced gravity fields exhibited similar columnar nondendritic grain structures.
(2) High centrifugal pressure shifted the corresponding eutectic point to the higher Al contents. As a result, the FCC phase content decreased, and that of the B2 phase increased.
(3) The B1 phase content increases in the system eventually due to the solid phase transition under enhanced gravity, and the main contribution of the increase amount of BCC structure phases comes from the B2 phase.
(4) The mass transfer enhanced by the enhanced gravity promoted element diffusion and phase enrichment. The Al and Ni contents in the B2 phase increased obviously with higher acceleration value and Cr contents in the B1 phase increased slightly with higher acceleration value, while Co, Cr, and Fe contents increased in the FCC phase.
(5) At acceleration 360 g, the compressive strength and plasticity of the corresponding sample were significantly enhanced and reached 2845 MPa and 36.4%, respectively.

## Figures and Tables

**Table 1:** Chemical composition of the initial Al_0.9_CoCrFeNi alloy (mass %).

Al	Co	Cr	Fe	Ni
9.5	23.2	20.7	21.8	24.8

**Table 2:** The chemical composition of the Al_0.9_CoCrFeNi alloy (mass %).

Al_0.9_CoCrFeNi	Regions	Chemical Composition				
		Al	Co	Cr	Fe	Ni
*G* = 1	A (BCC structure phases)	10.58	21.57	20.61	20.09	23.17
	B (FCC structure phases)	7.13	23.88	22.89	22.50	23.60
	B2 phase	11.35	20.60	19.93	18.73	24.00
	B1 phase	8.41	24.31	22.54	23.92	20.82
*G* = 360	C (BCC structure phases)	16.36	22.40	10.20	14.91	33.07
	D (FCC structure phases)	3.54	24.54	25.06	26.78	17.40
	B2 phase	19.48	18.14	5.80	16.54	35.91
	B1 phase	7.41	34.61	22.81	10.24	24.93

**Table 3:** Mechanical properties of the Al_0.9_CoCrFeNi alloy samples prepared at acceleration 1 g, acceleration 140 g, acceleration 210 g, and acceleration 360 g.

Different Acceleration Value	σ (MPa)	ε (%)
acceleration 1 g	2106	38.2
acceleration 140 g	2324	27.9
acceleration 210 g	2595	34.9
acceleration 360 g	2845	36.4

**Table 4:** Mechanical properties of the Al_x_CoCrFeNi alloys (x = 0.5~0.9) reported in the literature and this work.

Samples	*σ_max_*	*εp*	Phase	Ref.	Preparation
	(MPa)	(%)			
Al_0.9_CoCrFeNi	2845	36.4	FCC+BCC	Our work	Enhanced gravity solidification
AlCoCrFeNi	2864	22.7	BCC	[33]	Normal gravity solidification
AlCoCrFeNi	2670	22.5	BCC	[34]	Normal gravity solidification
AlCoCrFeNi	1668	26.4	FCC+BCC	[30]	Additive manufacturing
AlCoCrFeNi	1447	14.5	FCC+BCC	[35]	Additive manufacturing
Al_0.75_CoCrFeNi	1950	33.2	FCC+BCC	[27]	Suction-casting
Al_0.75_CoCrFeNi	1870	22	FCC+BCC	[36]	Combustion synthesis under enhanced gravity
Al_0.75_CoCrFeNi	1193	27.2	FCC+BCC	[37]	Normal gravity solidification
Al_0.5_CoCrFeNi	1750	32	FCC+BCC	[38]	Combustion synthesis under enhanced gravity
